# ALS-linked FUS mutants affect the localization of U7 snRNP and replication-dependent histone gene expression in human cells

**DOI:** 10.1038/s41598-021-91453-3

**Published:** 2021-06-04

**Authors:** Ankur Gadgil, Agnieszka Walczak, Agata Stępień, Jonas Mechtersheimer, Agnes Lumi Nishimura, Christopher E. Shaw, Marc-David Ruepp, Katarzyna Dorota Raczyńska

**Affiliations:** 1 Department of Gene Expression, Institute of Molecular Biology and Biotechnology, Faculty of Biology, Adam Mickiewicz University, Poznan, Poland; 2 Center for Advanced Technology, Adam Mickiewicz University, Uniwersytetu Poznańskiego 10, 61-614 Poznan, Poland; 3 UK Dementia Research Institute Centre at King’s College London, Institute of Psychiatry, Psychology and Neuroscience, King’s College London, Strand, London, WC2R 2LS UK; 4 Centre for Brain Research, University of Auckland, 85 Park Road, Grafton, Auckland, 1023 New Zealand

**Keywords:** Neuroscience, Cell death in the nervous system, Cellular neuroscience, Diseases of the nervous system, Molecular neuroscience, Molecular biology, Non-coding RNAs, RNA-binding proteins, Biochemistry, Proteins, Nuclear transport, Protein transport

## Abstract

Genes encoding replication-dependent histones lack introns, and the mRNAs produced are a unique class of RNA polymerase II transcripts in eukaryotic cells that do not end in a polyadenylated tail. Mature mRNAs are thus formed by a single endonucleolytic cleavage that releases the pre-mRNA from the DNA and is the only processing event necessary. U7 snRNP is one of the key factors that determines the cleavage site within the 3ʹUTR of replication-dependent histone pre-mRNAs. We have previously showed that the FUS protein interacts with U7 snRNA/snRNP and regulates the expression of histone genes by stimulating transcription and 3ʹ end maturation. Mutations in the *FUS* gene first identified in patients with amyotrophic lateral sclerosis (ALS) lead to the accumulation of the FUS protein in cytoplasmic inclusions. Here, we report that mutations in FUS lead to disruption of the transcriptional activity of FUS and mislocalization of U7 snRNA/snRNP in cytoplasmic aggregates in cellular models and primary neurons. As a consequence, decreased transcriptional efficiency and aberrant 3ʹ end processing of histone pre-mRNAs were observed. This study highlights for the first time the deregulation of replication-dependent histone gene expression and its involvement in ALS.

## Introduction

During the evolution of eukaryotes, molecular mechanisms were established to ensure accurate assembly of newly replicated DNA into chromatin. Therefore, in the S phase of the cell cycle, DNA synthesis is tightly coupled with histone protein synthesis. These two processes are finely balanced, as any disturbance may result in dysregulation of gene expression, cell cycle arrest, and chromosome instability, which may lead to developmental failure^1^. This coordination is most sophisticated in higher eukaryotes.

The core histones H2A, H2B, H3, and H4 and the linker histone H1 are replication-dependent histones (RDH) and are responsible for DNA packaging. Their expression is strongly upregulated during the G1*/*S phase transition, with their mRNA levels increasing by ∼ 35-fold in S phase due to activated transcription, efficient 3ʹ end processing and enhanced transcript stability^2,3^. At the end of the S phase, the availability of histones is repressed because an excess could be harmful to the cells^2^. In metazoan cells, replication-dependent histone mRNAs are not polyadenylated, and 3ʹ end processing relies only on a single cleavage event that is carried out by the endonuclease CPSF73 and mediated by a subset of specialized factors that recognize specific elements on the nascent transcripts^4–6^. Additionally, several of these factors are cell cycle-regulated, and their highest activity is in the S phase of the cell cycle^7–9^.

One of the key factors required for replication-dependent histone pre-mRNAs is the U7 small nuclear ribonucleoprotein (U7 snRNP). The U7 snRNP consists of the U7 snRNA and five Sm proteins that are shared with spliceosomal snRNPs (SmB/B', SmD3, SmE, SmF and SmG) and two unique Sm-like proteins: Lsm10 and Lsm11^6,10,11^. Initially identified by Birnstiel and colleagues in 1983 in *Xenopus* oocytes^12^, the U7 small nuclear RNA (U7 snRNA) encompasses 63 nucleotides in humans and is synthesized by RNA polymerase II (RNAP2)^12–14^ with approximately 10^3^–10^4^ copies present in mammalian cells. The U7 snRNA 5ʹ end is complementary to the histone downstream element (HDE), which is located downstream of the cleavage site within the 3ʹUTR of histone pre-mRNAs. Binding of the U7 snRNP to the HDE is crucial for correct 3ʹ end processing^13,15,16^.

Recently, we discovered that fused in sarcoma (FUS) is a linking factor that coordinates both RDH gene transcription and the 3ʹ end processing of their pre-mRNAs^17^. During the S phase of the cell cycle, FUS interacts with U7 snRNP and binds to histone promoters to enhance the binding of RNAP2 to histone genes^17^. FUS is predominantly nuclear, is ubiquitously expressed and is capable of binding to DNA and RNA. This protein plays a role in genomic maintenance and DNA recombination^18–20^, in addition to regulating RNA metabolism and processing. These processes include transcription, splicing, alternative splicing, miRNA biogenesis and nucleocytoplasmic shuttling of mRNAs^20–24^. In human neurons, FUS is located at synaptic sites along dendrites and has an important function in transporting mRNA for local translation, as well as its involvement in synaptic plasticity^25,26^.

In 2009, mutations in the *FUS* gene were identified in patients with amyotrophic lateral sclerosis (ALS)^27,28^. ALS is a chronic, progressive and uniformly fatal neurodegenerative disorder affecting motor neurons in the brain and spinal cord. Most FUS-linked ALS-causing mutations are missense mutations clustered in the highly conserved C-terminal nuclear localization signal (NLS) that disrupt the interaction with Transportin, which is required for nuclear import^29^. Mutations in the NLS can abolish or significantly reduce nuclear import of FUS, leading to its accumulation in the cytoplasm. Cytoplasmic inclusions containing FUS are found in neurons and glial cells in the brain and spinal cord of ALS patients^20,22,23,28–32^. In some cells, intranuclear inclusions containing FUS have also been described^33^. Splicing dysregulation and transcription impairment have been reported as a consequence of disruptions in FUS in mammalian cells as well as in ALS patients’ fibroblasts^34–36^. In addition, studies suggest that FUS aggregates affect local mRNA translation at the synapse and contribute to synaptic loss in ALS motoneurons^33^. Interestingly, FUS cytoplasmic aggregates sequester U snRNPs (U1 snRNPs, U2 snRNPs, U1 snRNA, U11 snRNA and U12 snRNA), FUS-binding proteins (hnRNP A1, hnRNP A2, and SMN), and poly(A) mRNAs^37–44^.

Here, we show that U7 snRNP is also trapped in cytoplasmic aggregates along with ALS-linked FUS mutants in cellular models and primary neurons. As a consequence of this mislocalization, we observed decreased transcriptional efficiency of RDH genes and aberrant 3ʹ end processing of their pre-mRNAs in SH-SY5Y cells. These changes can lead to histone instability and increased toxicity in neurons and motor neurons and thus can be involved in the pathogenesis of FUS-ALS.

## Results

### ALS-linked FUS mutations affect the cellular localization of U7 snRNA/snRNP

**Figure 1:**
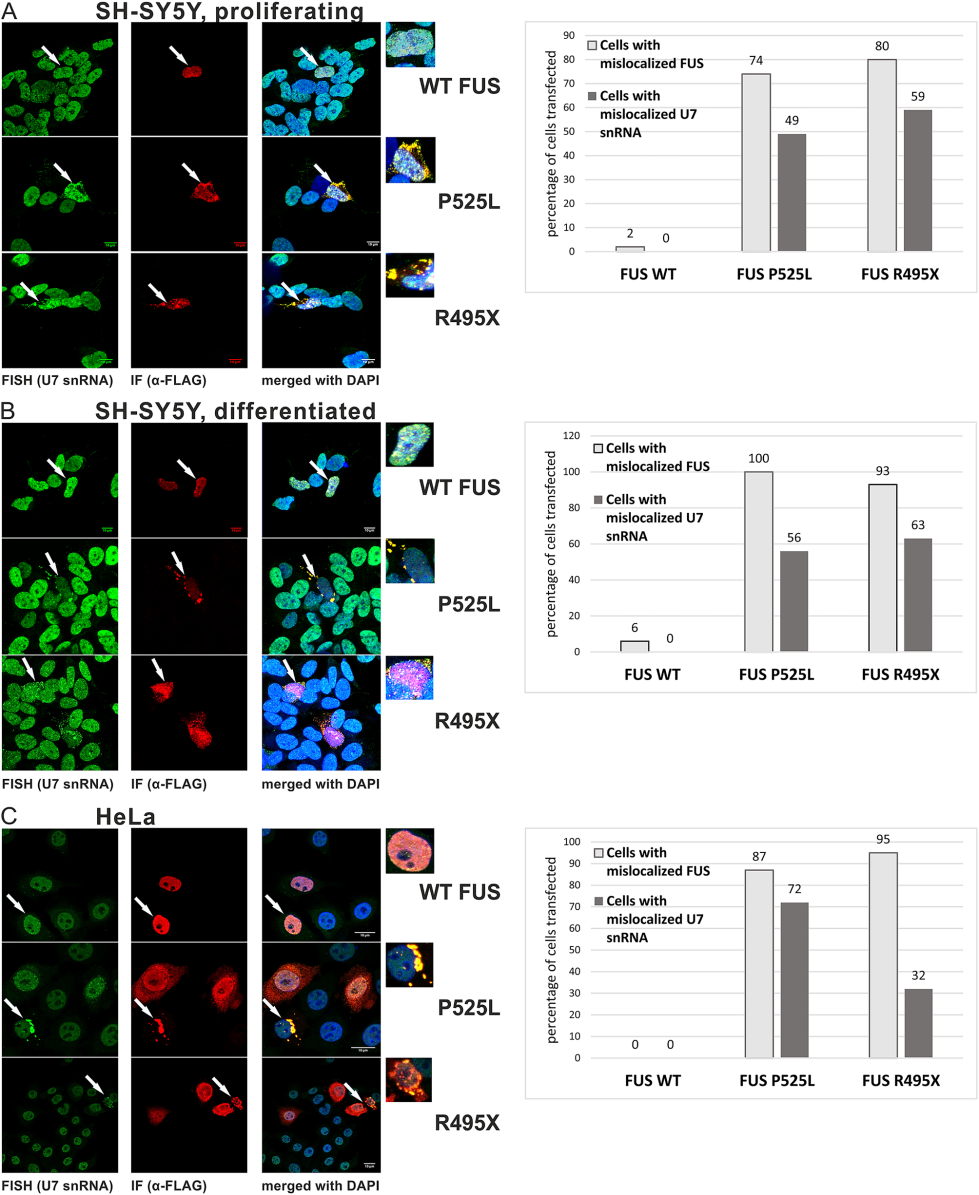
Localization of FUS and U7 snRNA in cells transfected with plasmids carrying FUS WT and ALS-linked FUS mutations. Fluorescent in-situ hybridization (FISH) using a probe against U7 snRNA in combination with immunofluorescence (IF) using anti-FLAG antibodies was performed in SH-SY5Y FUS KO (**A**,**B**) and HeLa FUS KO (**C**) cells transfected with FLAG-tagged FUS. DAPI was used for nuclear staining. Graphs show the percentage of transfected cells with mislocalization of FUS and U7 snRNA. WT FUS—cells transfected with plasmids encoding the wild-type *FUS* gene. P525L and R495X—cells transfected with plasmids encoding the *FUS* gene with P525L and R495X mutations.

FUS knockout SH-SY5Y cells (FUS KO) were transiently transfected with vectors encoding ALS-linked FLAG-tagged wild-type FUS cDNA or cDNA from the ALS-linked FUS mutants FUS-P525L and R495X. Both mutations lead to a severe ALS clinical phenotype and a striking cytoplasmic accumulation of FUS^29,31^. Experiments were performed using both proliferating cells and cells differentiated to neuron-like cells by the addition of retinoic acid^45^. Two days after transfection, the cells were fixed and subjected to immunofluorescence (IF) and fluorescence in situ hybridization (FISH). As shown in Fig. 1, wild-type FUS was located in the nucleus in the proliferating and differentiated cells transfected with wild-type FUS (Fig. 1A,B, upper panels). In contrast, in the P525L- and R495X-transfected cells, the ALS-linked FUS proteins were localized in cytoplasmic aggregates. These cytoplasmic aggregates also contained U7 snRNA (Fig. 1A,B, middle and lower panel). We repeated this experiment in primary neurons isolated from rat brain, transfected with the FUS mutants P525L. In line with the previous results obtained from SH-SY5Y cells, we again observed that U7 snRNA was recruited into cytoplasmic aggregates along with the mutant FUS (Supplementary Fig. S1A). These experiments were also conducted in FUS-knockout HeLa cells, and similar results were obtained (Fig. 1C).

Based on these results, we investigated whether the ALS-linked FUS mutations are associated with the mislocalization of other U7 snRNP components. To answer this question, we analyzed the localization of Lsm11 in cells transfected with FUS mutants P525L or R495X and compared with that in cells transfected with wild-type FUS. Lsm11 is a unique protein for the U7 snRNP complex. When transfected with wild-type FUS, Lsm11 was detected as a focus inside the nucleus of differentiated SH-SY5Y FUS KO, primary neurons and HeLa FUS KO cells (Supplementary Figs.S1B, S2A,B, upper panels). However, in cells transfected with mutant FUS, Lsm11 was recruited into cytoplasmic aggregates along with FUS (Supplementary Figs. S1B, S2A,B, middle and lower panel).

Taken together, our data suggest that mutations in FUS lead to mislocalization of the U7 snRNP complex to cytoplasmic aggregates.

### Mislocalization of FUS and U7 snRNA/snRNP leads to deregulated expression of RDH genes

**Figure 2:**
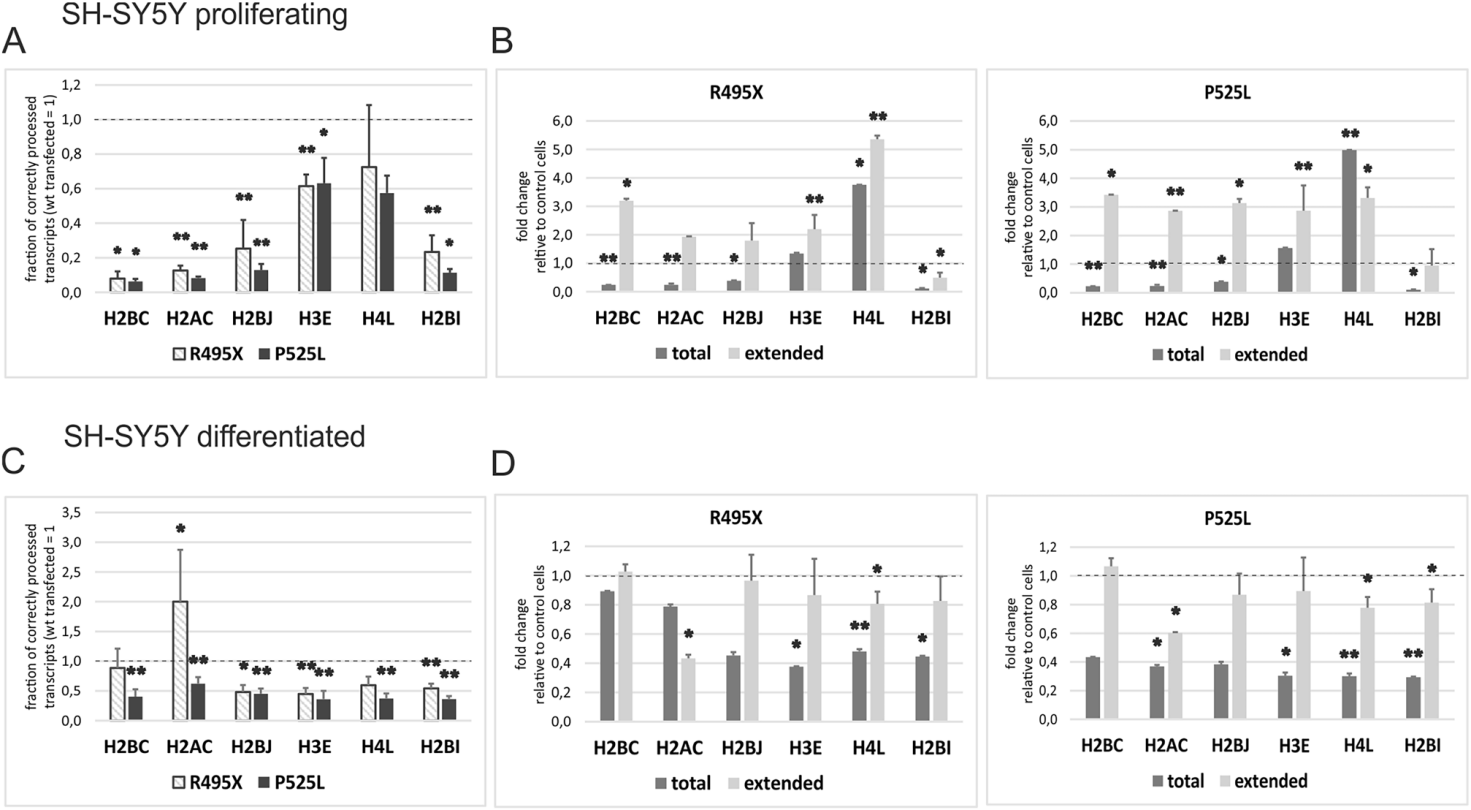
Replication-dependent histone gene expression in cells transfected with plasmids carrying FUS WT and ALS-linked FUS mutations. (**A**,C) The fraction of correctly cleaved histone mRNAs in the proliferating (**A**) and differentiated (**C**) cells transfected with the FUS mutants compared to the cells transfected with wild-type FUS displayed as the ratio of total to unprocessed (extended transcripts). (**B**,**D**) Fold enrichment of total and extended transcripts in the cells transfected with the FUS mutants compared to the cells transfected with wild-type FUS. H2A.Z was used as a reference gene. Error bars represent the SD of three biological replicates. P-values were calculated using Student's t-test, and the statistical significance is represented as follows: *P ≤ 0.05; **P ≤ 0.01.

Recently, we showed that FUS interacts with U7 snRNA/snRNP and participates in the 3ʹ end processing of replication-dependent histone pre-mRNAs. In cells with depleted FUS, we observed an elevated level of incorrectly processed, extended transcripts, which resulted from decreased processing efficiency^17^. To investigate the effect of mutant FUS on histone pre-mRNA processing, we isolated RNA from SH-SY5Y FUS KO cells transfected with FUS (WT) and FUS mutants (R495X and P525L) and analyzed the processing efficiency of different histones by RT-qPCR. The fraction of correctly cleaved histone mRNAs is displayed as the ratio of total to incorrectly processed (extended) transcripts, which represents the apparent processing efficiency, as described in^17^. The replication-independent histone gene H2A.Z, which undergoes cleavage and polyadenylation, was used as a reference. Indeed, compared to the cells transfected with WT FUS, the proliferating SH-SY5Y FUS KO cells transfected with the ALS-linked FUS mutants showed a significantly affected processing efficiency in the majority of histones analyzed (Fig. 2A). This effect resulted from an elevated level of extended transcripts; concomitantly, the level of total transcripts was significantly downregulated (Fig. 2B). Unfortunately, the low transfection efficiency prohibited the analogous experiment in primary neurons.

We have previously reported that decreased levels of total histone transcripts correlate with weaker binding of FUS to histone promoters and diminished levels of RNAP2 on histone genes^17^. To investigate whether the ALS-linked FUS mutations affect the activation of RDH gene transcription, we performed a ChIP assay in proliferating SH-SY5Y—FUS KO cells transfected with WT FUS or the P525L and R495X mutants. The RNAP2 occupancy on the promoter region/5ʹUTR, open reading frame (CDS) and 3ʹUTR of three RDH genes, H2AC, H2BJ and H4J, was analyzed by qPCR, as previously described^17^. The background signal was removed by subtracting the signals of control cells (non-transfected), and the fold enrichment of precipitated material from the cells transfected with the ALS-FUS mutants compared to the cells transfected with WT FUS was calculated. As shown in Supplementary Figure S3, mutations in FUS led to decreased RNAP2 occupancy in almost all three regions in the three histones analyzed. Collectively, our data suggest that mutations in FUS lead to inhibition of replication-dependent histone gene transcription in proliferating cells due to weak loading and binding of RNAP2 on histone genes.

Interestingly, no obvious impairment in 3ʹ end maturation was observed in the neuron-like SH-SY5Y FUS KO differentiated cells (Supplementary Fig. S3). Although “processing efficiency” was significantly downregulated in the mutant cells, this finding is probably due to inhibited transcription rather than affected 3ʹ end processing (Fig. 2C). Terminally differentiated cells were shown to synthesize only polyadenylated histone transcripts in a process that does not require U7 snRNP^46^. Therefore, the results in neuronal differentiated cells are due to accumulation of mature polyadenylated histone mRNAs instead of the incorrectly processed transcripts (Fig. 2D). We conclude that the “reduced processing efficiency” resulted from decreased levels of total pre-mRNAs and reduced transcription. This result may be due to the loss of nuclear function of the transcriptional activator FUS.

## Discussion

### Biological consequences of deregulation of RDH gene expression

We previously found that FUS is important in replication-dependent histone gene expression^17^. Here, we further investigated how ALS-linked mutations in the *FUS* gene can lead to disruption of the transcriptional activity of FUS and mislocalization of U7 snRNA/snRNP in cytoplasmic aggregates. As a consequence, we observed decreased transcriptional efficiency and aberrant 3ʹ end processing of histone pre-mRNAs, which is similar to what we observed in inducible HeLa FUS knockdown cells^17^. This study is the first to report deregulation of replication-dependent histone gene expression in the pathogenesis of ALS.

The involvement of FUS in DNA structure and chromosome maintenance has been previously shown^18–20^. FUS is involved in the formation of D-loops that are present during homologous recombination, DNA repair and telomeres. This protein is important for the induction of the DNA damage response and recruitment of the repair complex at the sites of double-stranded breaks (DSBs)^18,20,24^. FUS knockout mice are characterized by high levels of genome instability, enhanced sensitivity to ionizing radiation and increased chromosomal disturbances in premeiotic spermatocytes^47,48^. Accumulated DNA damage caused by a reduction in nonhomologous end joining (NHEJ)-mediated DSB repair was observed in neurons of the postmortem motor cortex from patients with FUS-ALS^49^. The increased DNA damage was also suggested to cause neurodegeneration in motoneurons reprogrammed from induced pluripotent stem cells (iPSCs) derived from FUS-ALS patients^33^. Naumann and collaborators even proposed that DNA damage is an upstream event that enhances aggregate formation, cytoplasmic localization of FUS and neurodegeneration^50^. Taken together, increased DNA damage in human ALS patients harboring FUS mutations together with disrupted histone synthesis can cause genome instability and may be the molecular mechanisms underlying altered glial cell or motor neuron homeostasis in ALS.

To test the effect of the ALS-linked mutations of FUS on transcriptional efficiency and 3ʹ end processing of histone pre-mRNAs, we transiently expressed FUS in SH-SY5Y and HeLa cell lines, in both wild-type and FUS knockout cell lines. However, probably due to the high expression of endogenous FUS in wild-type cells, the effect that we observed was more prominent in the FUS KO cells transfected with the ALS-linked FUS mutants. Notably, due to the limited transfection efficiency, the result is representative of cells that contain exogenous FUS and of cells that do not contain FUS. Therefore, the effect of the ALS-linked FUS mutations on RDH gene expression might still be underestimated.

Higelin and colleagues have suggested that in iPSCs and motoneurons with ALS-linked FUS mutations, additional external factors such as DNA damage or hyperosmolar stress can induce changes in FUS mislocalization and stress granule formation. This change was accompanied by impaired DNA damage repair since FUS is a component of the DSB repair machinery. Interestingly, the researchers observed that the accumulation of DNA damage and the cellular response to DNA damage stressors were more pronounced in the postmitotic mutant FUS motoneurons than in the dividing pluripotent cells. Therefore, maturation, accumulation of DNA damage foci and later events in older motoneurons were supposed to increase the size and toxicity of FUS inclusions and finally induce neurodegeneration^33^. Under natural conditions, in motoneurons in the patient’s brain, these factors might also enhance the negative effect of mutated FUS on U7 snRNA/snRNP function and RDH gene expression.

### Biological consequences of U7 snRNA/snRNP mislocalization

As shown in Fig. 1 and Supplementary Figure S1, in HeLa FUS KO cells, in proliferating and differentiated SH-SY5Y FUS KO cells, and in primary neurons, the ALS-linked FUS mutations cause mislocalization of FUS and U7 snRNA in cytoplasmic aggregates. In the proliferating cells, this change led to deregulation of histone gene expression (transcription and processing of pre-mRNAs). As discussed above, this phenomenon can result in abrogated histone synthesis and, as a consequence, can cause genome instability. In contrast, in the differentiated neurons, replication-dependent histone gene expression was almost silenced ^46^ and necessary histones are eventually produced from extended, polyadenylated transcripts. For this reason, the effect of the ALS-linked FUS mutations on the unique process of 3ʹ end maturation of RDH pre-mRNAs was not detectable (Fig. 2D) However, the U7 snRNP in these cells was still present (Figs. 1B and S1A). The question arises as to which biological pathway is then affected by the mislocalized U7 snRNA/snRNP. The answer may shed new light on the molecular mechanisms underlying altered motor neuron homeostasis in ALS patients as well.

In summary, together with major and minor spliceosomal snRNPs, U7 snRNP is another snRNP in the cell, whose activity is affected by ALS-linked FUS mutations. As these snRNPs play different functions, the potential defects in ALS patients are expanded from splicing defects to replication-dependent histone processing defects. In this report, we have also expanded the known defects in snRNP/snRNA metabolism.

## Materials and methods

### Cell culture and differentiation

SH-SY5Y and HeLa cells were grown in Dulbecco’s modified Eagle’s medium containing l-glutamine and 4.5 g*/*L glucose (DMEM; Lonza) and supplemented with 10% fetal calf serum (Gibco) and antibiotics (100 U*/*ml penicillin, 100 µg*/*ml streptomycin (Sigma-Aldrich)) at 37 °C in a humidified atmosphere containing 5% CO_2_. Rat primary cortical neurons were isolated from E18 pups and plated on 100 µg/ml PDL coated chamber slides (ibidi). Neurons were maintained in Neurobasal media supplemented with 1% Glutamax, 1% B27 supplement and 1% Penicillin/Streptomycin media (Life Technologies). SH-SY5Y and HeLa cells with FUS KO were prepared as described previously^39,51^. For cellular differentiation, SH-SY5Y FUS KO cells were subjected to retinoic acid (RA) treatment for a period of 10 days to transform them into neuron-like cells^52^. All-trans RA (USP, Tretinoin, 1674004) at a final concentration of 75 µM was added to DMEM with 10% FBS. The media along with RA were replaced every 3 days. After 10 days, fresh media without RA were replaced just before transfection. The differentiation efficiency was analyzed as follows: (i) level of differentiation markers: the transcription factor MYC and Growth Associated Protein 43 (GAP43) were tested by RT-qPCR (Supplementary Fig. S4A); (ii) actin staining was performed to show neuron-like cells (Supplementary Fig. S4B); and (iii) expression of Microtubule Associated Protein 2 (MAP2) in differentiated cells was confirmed by Western blots (Supplementary Fig. S4C).

### Plasmid and cell transfection

The plasmids used in the experiments are based on the pCDNA vector encoding cDNA of the FUS WT plasmid, the FUS R495X plasmid where the amino acid sequence is truncated after arginine at position 495, and the FUS P525L plasmid where proline is mutated to leucine at position 525. The plasmids used for the transfection of SH-SY5Y and HeLa cells were kindly obtained from Don Cleveland’s group (described in^37^), while the plasmids used for the transfection of rat cortical neurons were described in^39^. Transient transfections were performed with VIROMER RED (Lipocalyx) or Lipofectamine 2000 according to the manufacturer’s instructions. Cells were harvested 48 h after transfection for further experiments.

### Immunofluorescence and fluorescence in situ hybridization

SH-SY5Y FUS KO cells, HeLa FUS KO cells and primary neurons were grown on chambered coverslips. SH-SY5Y FUS KO cells and HeLa FUS KO cells were transfected with 250 ng of plasmid DNA (FUS WT, FUS P525L, FUS R495X), using VIROMER RED (Lipocalyx). Forty-eight hours after transfection, the cells were fixed with 4% PFA and permeabilized in 1× PBS pH 7.0 + 0.5% Triton X-100 (PBS-T). The rat primary cortical neurons were transfected on DIV7, with 175 ng of plasmid DNA pcDNA6F-FUS-wt and pcDNA6F-P525L using Lipofectamine 2000 following supplier’s recommendations. Neurons were fixed on DIV9 with 4% PFA for 15 min at room temperature and pre-treated with 70% ethanol for 2 min at − 20 °C and finally stored in 100% ethanol at − 80 °C until staining. On the day of the staining, cells were washed twice with 70% ethanol for 2 min and three time with PBS for 5 min. Proceeding the protocol, the cells were incubated with blocking solution (1% BSA in PBS, 200 mM ribonucleoside vanadyl complex) for 30 min at RT. Primary antibody incubation for FUS was either with the mouse anti-FLAG antibody (1:200, Merck F3165) or anti-FUS antibody (Santa Cruz, SC 47711) for 1 h at RT. Cells were washed in blocking solution followed by secondary anti-mouse Alexa Fluor 555 antibody (Thermo Fisher Scientific A21422) or Alexa Fluor 546 (Thermo scientific A11030) in blocking solution for 45 min RT. Following immunofluorescence, the cells were washed 3 times with PBS and subsequently post-fixed with 4% PFA for 5 min at RT. The cells were then washed twice with 2× SSC (300 mM NaCl, 30 mM sodium citrate pH 7.0) and incubated in pre-hybridization buffer (15% formamide, 10 mM sodium phosphate, 2 mM RVC in 2× SSC) for 10 min at RT. U7 snRNA-specific probe was prepared with RNA Labeling Kit–488 (BaseClick BCK-RNA488-10). Briefly, in vitro transcription was carried out with the use of a plasmid template (full-length U7 snRNA complementary sequence cloned into the pBS plasmid) and the addition of 5-ethynyl-UTP. All the steps of in vitro transcription and labeling were performed according to the manufacturer’s protocol. The probe was then diluted to 0.75 ng/μl in hybridization buffer (15% formamide, 10 mM sodium phosphate, 10% dextran sulfate, 0.2% BSA, 0.5 μg/μl salmon sperm DNA, 0.5 μg/μl *Escherichia coli* tRNA, 2 mM RVC in 2 × SSC) and hybridized to the cells overnight at 37 °C. The following day the cells were washed twice in pre-hybridization buffer for 30 min at 37 °C and three times for 10 in high stringency buffer (20% formamide, 2 mM RVC in 0.05× SSC). Subsequently, the cells were washed three times in 2× SSC for 2 min at RT. Washing steps were performed to remove unbound probes, and the slides/coverslips were mounted with ProLong Gold aqueous DAPI containing mounting medium or using Vectashield containing DAPI. For detection of LSM11, the cells were fixed, washed and blocked as described above. Rabbit anti-LSM11 antibody (1:200, Merck HPA039587) was applied followed by secondary anti-rabbit Alexa Fluor 555 antibody or anti-rabbit Alexa Fluor 546 antibody.

The images for SH-SY5Y FUS KO and HeLa FUS KO cells were acquired with a confocal scanning microscope (Nikon A1Rsi) using a 100×/1.4 or 63×/1.4 oil-immersion objective. Excitation was achieved with an argon laser at 488 nm (6-FAM: U7 snRNA) and with a diode laser at 561 nm (Alexa Fluor 555: FUS, Lsm11), 405 nm (DAPI). Images were analyzed using ImageJ open source software. Images for the rat cortical neurons were acquired using the VT-iSIM microscope (Nikon) using a 20×/dry or 100×/1.49 NA oil immersion lens. Deconvolution was performed with the NIS-Elements AR software (Ver 5.01) using the Richardson/Lucy algorithm with 15 iterations.

### RNA isolation, cDNA preparation and qPCR

For RNA isolation, a Quick-RNA MiniPrep Kit (Zymo Research, R1055) was used according to the manufacturer’s protocol. For cDNA preparation, 0.5 µg of random hexamer primer was used for 0.5–3 µg of RNA in a total volume of 25 µl. The samples were denatured at 65 °C for 5 min, and then, master mix consisting of 1× first strand buffer, 0.5 µM dNTPs, 1 µl of RNasin (40 U/µl stock concentration), 5 mM DTT and 200 U Super Script III Reverse transcriptase was added. The reaction was incubated for 1 h at 50 °C followed by a 10 min incubation at 75 °C. For qPCR, 1 µl of 3–5× diluted cDNA template, 0.2 µM primer mix (forward + reverse) and 5 µl of SYBR Green PCR master mix (Applied Biosystems) were added in a 10 µl reaction with the following conditions: denaturation for 10 min at 95 °C, followed by 40 cycles of 95 °C for 15 s and 60 °C for 1 min (Applied Biosystems QuantStudio 7 Flex).

### Western blotting analyses

For immunodetection, proteins were separated by SDS–polyacrylamide gel electrophoresis (PAGE), transferred to polyvinylidene difluoride (PVDF) membranes (Millipore), blocked with 5% skim milk diluted in PBS-T and then incubated for 1.5 h at room temperature (RT) with anti-MAP2 (Abcam, 5392, 1:8000 dilution in PBS-T) and anti-actin (MP Biomedicals, 691001, 1:50,000 dilution in PBS-T) primary antibodies. After 3 washes with PBS-T, the membrane was incubated for 1 h at RT with species-specific horseradish peroxidase (HRP)-coupled secondary antibody (Santa Cruz, goat anti-mouse, SC2005, 1:3000 dilution in PBS-T) followed by 3 washes as before. The signal was detected using the enhanced chemiluminescence method (ECL, GE Healthcare).

### Chromatin immunoprecipitation

Neuroblastoma SH-SY5Y FUS KO cells, control cells (non-transfected) and cells transfected for 48 h with FUS WT, FUS R495X and FUS P525L plasmids were trypsinized, washed with PBS and crosslinked using 10 ml of 1% formaldehyde for 10 min at RT. The crosslinking reaction was stopped by adding 800 µl of 1 M glycine and incubating for 3 min at RT with gentle swirling. The cells were centrifuged at 1000*g* for 1 min at 4 °C, and the pellet was washed with 1X PBS and then lysed in lysis buffer (72.3 mM NaCl, 5 mM EDTA pH 8.0, 0.5% NP-40, 50 mM Tris pH 8.0, 1X EDTA-free protease inhibitor (Roche)), pipetting to make a homogenous suspension. After lysis, the cells were centrifuged at 12,000*g* for 1 min at 4 °C, and the pellet was resuspended in 1 ml of sonication buffer (1% SDS, 10 mM EDTA pH 8.0, 50 mM Tris pH 8.0 and 1 protease inhibitor cocktail tablet/10 ml buffer). The cells were sonicated for 30 cycles at high intensity with 30 s ON/30 s OFF at 4 °C using a Bioruptor Plus sonicator (Diagenode) to generate fragments of DNA between 200 and 700 bp. The sizes of the fragmented DNA were verified by agarose gel electrophoresis. Before agarose gel analysis, the samples were treated with 1 U of RNase A (Thermo Scientific, EN0601) at 37 °C for 30 min. Next, 20 ng of proteinase K was added, and samples were incubated for 2 h at 45 °C at 1000 rpm. After sonication, the cell debris was removed by centrifuging the cells at 11,200*g* for 10 min at 4 °C. The supernatant was transferred to a new tube; 10% of the supernatant was kept as input. One hundred microliters of solution was transferred to another tube and diluted in a 1:10 ratio using dilution buffer (1 mM EDTA, pH 8.0, 0.01% SDS, 1.1% Triton-X 100, 0.17 M NaCl) and further used as an IgG control. The remaining solution was also diluted in a 1:10 ratio using dilution buffer and further used for immunoprecipitation (IP). One microgram of IgG (Invitrogen 10500C) was added to the IgG control probe, while 5 µg of RNAP2 (Abcam, ab10332 and ab10338) was added to the sample tubes. For IP, 15 µl of Dynabeads Protein G (Life Technologies) was pre-blocked with 0.5% bovine serum albumin (BSA) by overnight incubation at 4 °C. Next, the samples were centrifuged at 3500 rpm for 20 min at 4 °C. After centrifugation, the top 90% of the solution was transferred to another tube for IP, and the bottom 10% was discarded. For IP, 190 μl of protein G beads was added into the solution and incubated for 6 h at 4 °C with head-to-tail rotation. Next, the samples were washed 6 times with lysis buffer and rotated for 3 min at 4 °C. After the samples were washed, the beads were resuspended in 1 ml of ice-cold Tris–EDTA (TE) buffer to make a slurry. TE buffer was removed, and the beads were retained by keeping the tubes on a magnetic rack. Two hundred microliters of elution buffer (1% SDS, 0.1 M NaHCO_3_) was added to the beads as well as to the input and incubated for 15 min at 1000 rpm at room temperature. The eluted solution was transferred to new tubes, and 8 μl of 5 M NaCl and 4 μl of 0.5 M EDTA, pH 8.0 were added to each sample and incubated overnight at 65 °C at 1000 rpm to de-crosslink the immunoprecipitated chromatin. The probes were further digested by adding 10 μg of RNase A and incubating at 37 °C for 30 min. Next, 8 μl of 1 M Tris-HC, pH 8.0, 4 μl of 0.5 M EDTA and 20 ng of Proteinase K (Thermo Scientific) were added, and the samples were incubated for 2 h at 45 °C at 1000 rpm. The purified DNA was then used for qPCR.

### Ethics statement

All experiments concerning laboratory animals were carried out following the ethical guidelines of the Animal research reporting of in vivo experiments (ARRIVE), UK Animals (Scientific Procedures) Act 1986, and were approved by the Kings College, London ethics review panel. We further confirm that all methods were carried out in accordance with relevant guidelines and regulations.

## Supplementary Information

Supplementary Information.
