# Supplementary material for: ALS-linked FUS mutants affect the localization of U7 snRNP and replication-dependent histone gene expression in human cells
Source: Sci Rep. 2021 Jun 4;11:11868. doi: 10.1038/s41598-021-91453-3 (PMC8178370; doi:10.1038/s41598-021-91453-3)
Supplement: Supplementary file 1 — Supplementary Information. [file 41598_2021_91453_MOESM1_ESM.docx]

**Supplementary Information for**

**Manuscript title**

ALS-linked FUS mutants affect the localization of U7 snRNP and replication-dependent histone gene expression in human cells

**Authors**

Ankur Gadgil^1,2^, Agnieszka Walczak^1^, Agata Stępień^1^, Jonas Mechtersheimer^3^, Agnes Lumi Nishimura^3^, Christopher E. Shaw^3,4^, Marc-David Ruepp^3^, Katarzyna Dorota Raczyńska^1,2^*

*To whom correspondence should be addressed:

**Katarzyna Dorota Raczynska**, Department of Gene Expression, Institute of Molecular Biology and Biotechnology, Adam Mickiewicz University, Poznan, Poland, phone: +48618291901,
e-mail: [doracz@amu.edu.pl](mailto:doracz@amu.edu.pl)

**This PDF file includes:**

Supplementary

Figures S1 to S4

Supplementary Figure 1


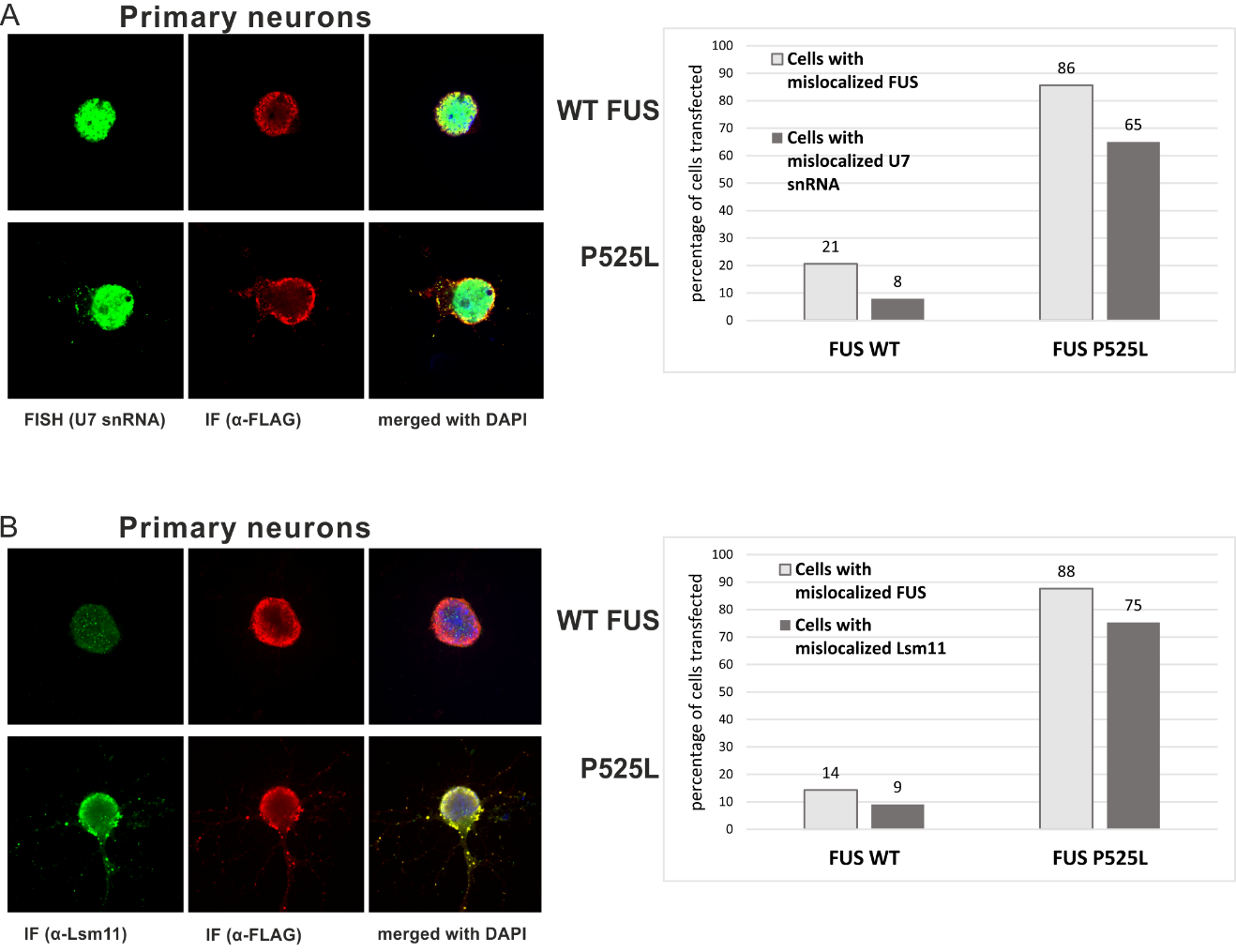


**Supplementary Figure S1**

**Localization of FUS, U7 snRNA and Lsm11 proteins**. (A) Fluorescent in-situ hybridization (FISH) using a probe against U7 snRNA in combination with immunofluorescence (IF) using anti-FLAG antibodies, and (B) IF using anti-FLAG and anti-Lsm11 antibodies was performed in primary neurons transfected with FLAG-tagged FUS. DAPI was used for nuclear staining. Graphs show the percentage of transfected cells with mislocalization of FUS and U7 snRNA or Lsm11. WT FUS – cells transfected with plasmids encoding the wild-type *FUS* gene, P525L – cells transfected with plasmid encoding the *FUS* gene with P525L mutation.

Supplementary Figure 2


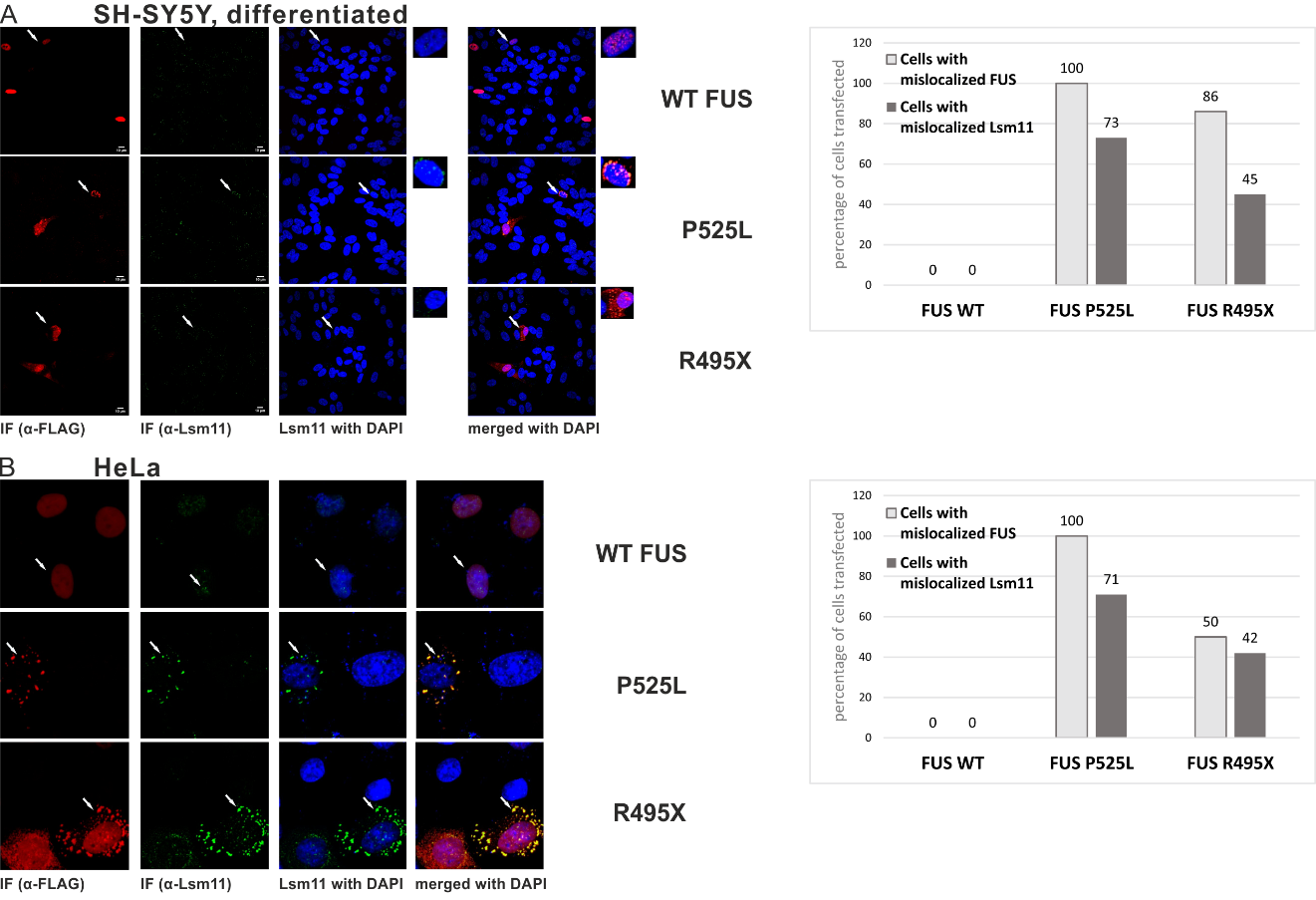


**Supplementary Figure S2**

**Localization of FUS and Lsm11 proteins**. IF using anti-FUS and anti-Lsm11 antibodies was performed on SH-SY5Y FUS KO (A) and HeLa FUS KO (B) cells. DAPI was used for nuclear staining. Graphs show the percentage of transfected cells with mislocalization of FUS and U7 snRNA. WT FUS – cells transfected with plasmids encoding the wild-type *FUS* gene, P525L and R495X – cells transfected with plasmid encoding the *FUS* gene with P525L and R495X mutations.

Supplementary figure 3


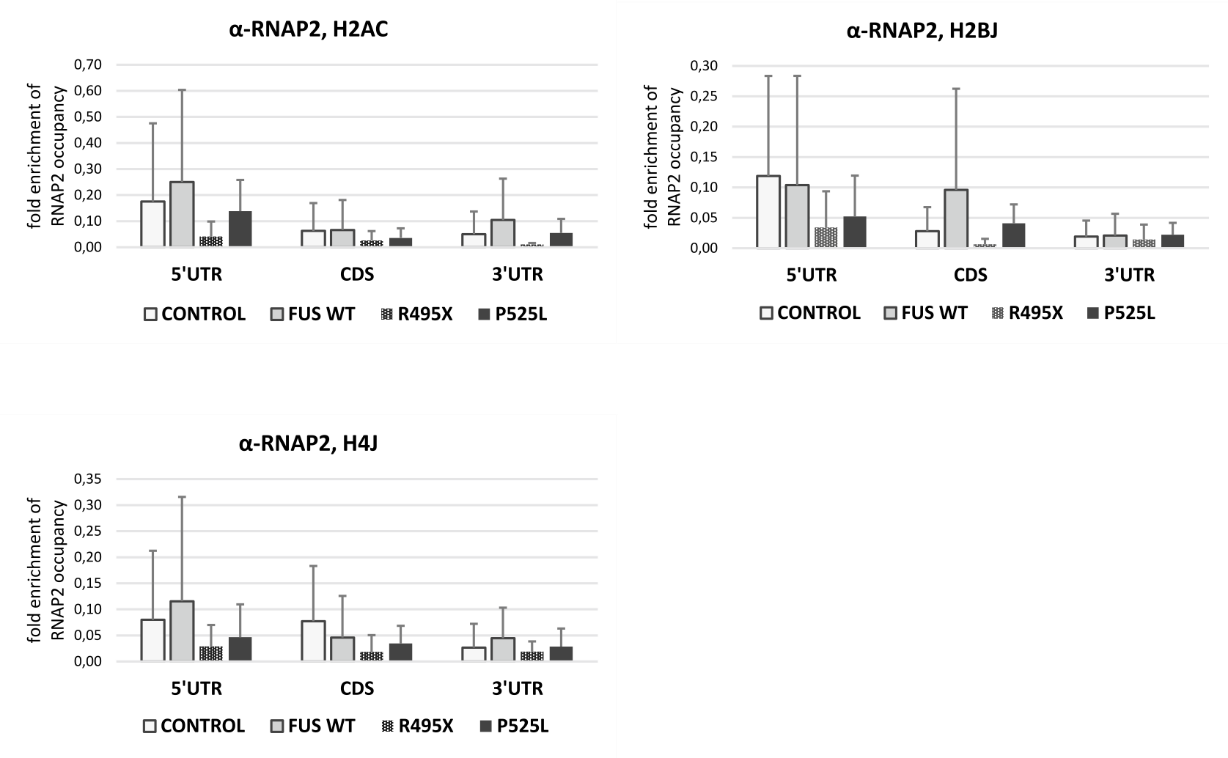


**Supplementary Figure S3**

**RNA Polymerase II (RNAPII) occupancy on histone genes.** ChIP-seq analyses followed by real-time quantitative PCR was performed on SH-SY5Y FUS KO cells (control, non-transfected) and the cells transfected with wild-type FUS (FUS WT), R495X and P525L FUS mutants. Charts represent the mean fold change value (n = 3). Graphs show RNAPII occupancy on the 5'UTR, CDS and 3'UTR for the replication-dependent histone genes H2AC, H2BJ, and H4J. The CT values from the non-transfected control cells were used to nullify the background. Input CT values were used as normalizers. Error bars indicate standard deviations (SD) of three biological replicates. P-values were calculated on percent of input values using Student’s t-test.

**Supplementary figure 4**


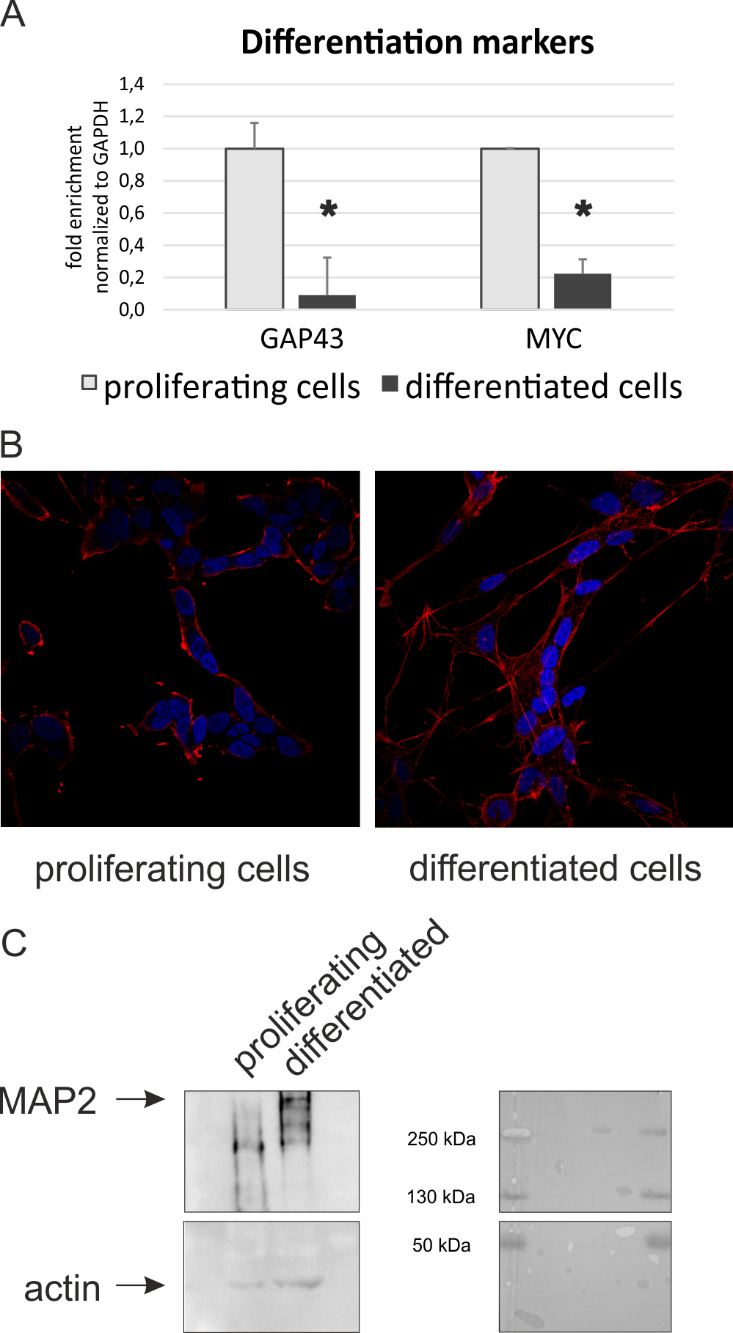


**Supplementary Figure S4**

**Confirmation of differentiation of SH-SY5Y FUS KO proliferating cells to neuron-like cells**.
A) The levels of the cellular proliferation markers growth-associated protein 43 (GAP43) and MYC were tested by real-time quantitative PCR. The marker level in proliferating cells was arbitrarily set at 1. Error bars indicate standard deviations (SD) of three biological replicates. P-values were calculated using Student’s t-test, and the statistical significance was represented as follows: *P ≤ 0.05.
B) Actin phalloidin staining showing dendrite and axon growth (observed in red) in SH-SY5Y cells differentiated into neuron-like cells; proliferating SH-SY5Y cells show reduced dendrite and axon growth. The nucleus was stained with DAPI.
C) Microtubule-associated protein 2 (MAP2), which is activated in cells differentiated into neuron-like cells, was detected by Western blots and immunodetection. Actin was used as a loading control
